# The Young Suicide

**Published:** 1863-12

**Authors:** 


### THE YOUNG SUICIDE.

WITHIN a month, a young collegian of twenty, who had the love and respect of his teacher and his classmates, for his diligence in his studies, his high classical position, and his generous nature, was found in his bed-room dead, the arteries of his arm having been severed, and his throat cut from ear to ear; an empty vial was found on the floor, labeled "Poison." A note on the stand read thus:

"Forgive me, my dearest parents, for this dreadful deed, but I am unworthy and unfit to live. May God forgive me! Farewell."

There are scores of such deaths every year, and the inquiry arises in every thoughtful mind: "What could have been the reason for such a terrible act?" This youth was the only child of rich parents; and the uninitiated vainly look around for an adequate motive for a deed so dreadful. A physician, especially a city physician, and more particularly the editor of a journal, treating of health and disease, is at no loss to unravel the mystery. The key to the sad history is found in the three facts, the victim was a man—young, unmarried. As an editor and a physician, we are constantly receiving letters like the following: "Can you save me from an ignominious grave? O God! that some one would give a cure for those unfortunates who have been so foolish. Will you, in God's name, give a cure in your next issue? and you will have the prayers of thousands." A day or two later came the following: "Dear Sir: In the midst of despair, I earnestly address you, thinking that perhaps my sorrow may by your skill be turned into joy. I have by indulgence ruined myself. I have applied to many without permanent benefit; hence the cause of despair. Now I feel the consequence of my crime to a very great extent mentally, in consequence of which I have been obliged to give up all employment and do nothing, and of late I am totally unfit for any thing. I have now before me a copy of your JOURNAL OF HEALTH, volume nine, for October, 1862. Hence I address you, hoping you may be able to suggest some means for regaining health and happiness. I feel as if I would surely die, if I do not obtain some active and immediate remedy." While supervising these lines for the press, a letter is received from a young gentleman of high position, untarnished reputation, and of high moral worth, active, energetic, and faithful in all the offices of trust in which he has been placed, and which he has never failed to fill with honor to himself, and credit to his friends. Such an one writes: "I am satisfied that hell is my portion in robbing nature, and robbing God, my Creator. My mind is giving way to despair. I am ashamed of myself before my fellow-men and before God. And if I am dealt with according to my deserts, I am doomed." The practical question, and one which very nearly concerns every parent who has an unmarried son over fifteen years of age, is, What can produce such states of mind? They arise in all cases from a vicious reading; from perusing books which are sent gratis and post-paid by cart-loads, to all parts of the country every year, through the agency of the newspapers, with advertisements headed in this wise—taking a city daily, at this present writing—and which are copied, for large "consideration," by the country press, (nor are all of our religious papers guiltless of the damning iniquity:) "To the Unmarried," "Marriage Guide," "Physiology," "The Benevolent Association," "Physiological Inquiries," "Young Man's Book," "Warning to Young Men," "Manhood," "Physical Debility," with a variety of other headings. These publications have the same aim, object, and end, and the midnight depravity which indites them stands out in every page. It is not necessary here to enter into minute details, but to make use of the general facts. The programme marked out by all of them is essentially the same. First, to pander to the vitiated curiosity of boys and youth, not only by the "pictorial illustrations drawn from life," but by speciousness of argument and reasoning and statements, to mislead the mind, inflame the imagination, corrupt the heart, and eventually degrade the whole character. It is an often remarked fact, that among the young gentlemen who attend a first course of medical lectures, there are a large number who imagine themselves the victims of each successive disease, as it is presented in course by the lecturer. And any person not versed in medicine can scarcely read any book on any disease, without beginning to imagine that he has more or less of its symptoms. In fact, medical biography abounds with notices of the deaths of men from the very diseases, the successful treatment of which made them famous; leaving us to suppose that imagination has something to do in causing, or at least in aggravating, some human maladies. It is not surprising, then, that youths in their teens, or just entering manhood should, in reading a treatise strongly depicting the ultimate effects of certain symptoms, alleged to be connected with certain conditions of the system, should run riot in their fears, and throw themselves helplessly into the hands of those who seem to know so much on the subject, and by their own accounts have had such remarkable success in their line. In every one of these books, without exception, certain symptoms are mentioned (not peculiar to any one disease, but common to a number, or which may exist, and if let alone, would in time disappear of themselves) as peculiar to a state of the system indicative of "a want of capabilities." Among these the most stereotyped are, dimness of vision, loss of memory, incapability of mental concentration, no steadiness of purpose, depression of spirits, etc. Then certain physical appearances are noted as corroborative of the existence of the malady in question. The youth, not having opportunities of comparing himself with others; not knowing that a good many of these very appearances are natural, and are not incompatible with perfect health, becomes alarmed, and in his fright appeals to the author of the book he has been reading, to save him by all means from the impending ruin and disgrace. A fee is extorted, which is up to the utmost ability of the victim to raise. Remedies are used. They do not change the condition of things; simply because the conditions are in many cases not unnatural; but the patient is made to believe that it is because the case is more desperate than was imagined, and that more powerful and more expensive remedies must be used. These are alike unavailing; meanwhile, weeks and months pass away; the victim has spent all the money he can "rake and scrape," "beg, borrow, or steal" literally, and then writes to some known physician in the strain of the letters already quoted, to make at least one more attempt at rescue; or if he does not this, he settles down in the despair which leads to suicide.

But there is sometimes a more dreadful ending, so far as mental and bodily suffering is concerned; we say more dreadful with design; for it is more so in proportion as it is more of a calamity to die on the rack than by a cannon-ball. When the sharper has obtained all the money possible from his victim, and wishes to get rid of him, he says in plain language: "There is no help for you but in marriage." But often this is an impossible remedy, and even if it were practicable, the patient has such a view of his condition, that he would consider it dishonorable and even infamous to impose himself upon a confiding woman. The sharper is prepared for this, and with practiced depravity, advises an illegal connection, not only as a test of capabilities, but as a remedy for certain symptoms observed to occur in the early morning, or at the close of certain natural actions. Human nature can seldom withstand the motives presented in cases like these. Six months ago a gentleman applied for advice under the following circumstances. He had been led, by reading a book on "physiology," to believe that in connection with certain practices a deplorable state of things was induced. He placed himself under the care of the writer of the book in question, and in two or three years had expended a considerable amount of money, without adequate results. He was then told that he must form a criminal liason, which he did with inconceivable loathing, and which he maintained until he found himself the victim of a degrading disease, showing itself on the face and hands. To escape the inquiries of relatives and friends, he left home and came to us, the embodiment of despair, the mind hopeless, the body ruined, the constitution a wreck. This disease the writer has never treated in a single case; and it is always turned over to other hands, the usual advice being, when there is any hope of restoration, to have recourse to the family physician at home, who would be more likely than any other to take a deep interest in the case, and exercise those sympathies which are so requisite under the circumstances.

Cases of this kind are of daily occurrence, and are constantly coming under the notice of city physicians, by hundreds and thousands every year. Some practical lessons of an importance which can not perhaps be over-estimated, may be drawn from this subject:

1. Allow no paper or magazine to enter your house which offers, by advertisement, to send any book on health and disease free of cost. No man can afford to print a book for nothing, and then to pay postage on it, unless he afterward finds his pay in the manner above described.

2. Let all parents encourage the early marriage of their sons; as soon after twenty-one as circumstances will permit; it is a less evil than to be exposed to the dangers above referred to, by putting it off to the more physiologically appropriate age of twenty-five.

3. Let no youth of intelligence ever consult a man at a distance, for the ailments which have been alluded to, or the supposed symptoms of impossible things. It is a thousand times better to consult the family physician at home; him you can trust with safety, as to body and reputation; a thing which is never to be done by men who send books free of charge, which treat of any form of disease.

As to the symptoms and debilitations of the early morning, and which have such a depressing influence on mind and body, second only to those of dyspepsia, nothing can be more certain than that there is no remedy, safe and efficient in drugs; but it must be sought in the diligent following out of some active industrial pursuit, force of will, and the cultivation of a high and manly moral power, which looks with angry and impatient contempt on all that is vicious, corrupting, and degrading, whether in deed or word or thought; this is the only efficient, the only infallible remedy, and is worthy of the mature reflection of every high-minded and generous-hearted youth. See the editor's book on "Sleep" for more full details.

